# Effects of Social Activities and APOE on Cognitive Decline in African Americans

**DOI:** 10.1093/geroni/igaf122.564

**Published:** 2025-12-31

**Authors:** Casey Brown, Neke Nsor, Kyle Bourassa, Lisa Barnes

**Affiliations:** Georgetown University, Washington, District of Columbia, United States; Georgetown University, Washington, District of Columbia, United States; Durham VA Medical Center, Durham, North Carolina, United States; Rush University Medical Center, Chicago, Illinois, United States

## Abstract

African Americans experience disproportionate rates of cognitive decline yet remain underrepresented in research on the biopsychosocial factors contributing to this decline. This study examines whether biological factors (APOE alleles) interact with social engagement (e.g., participation in social groups) to predict cognitive decline in African Americans. A sample of 734 African American adults from the Minority Aging Research Study (MARS), aged 65 and older and free of dementia at enrollment, completed annual cognitive assessments over 10 years. At baseline, participants underwent genetic testing and reported their engagement in social and cognitive activities. Structural equation modeling assessed the effects of APOE genotype, cognitive activities, and social activities on cognitive decline, along with their interaction effects over time. Results showed that APOE count influenced cognitive decline, such that a greater number of APOE4 alleles was associated with faster cognitive decline, while a greater number of APOE2 alleles was linked to slower cognitive decline. However, social activities did not interact with APOE count in predicting decline. Instead, APOE4 and social activities had independent, additive effects on cognitive decline. These findings underscore the role of social engagement in slowing cognitive decline in African Americans, regardless of APOE status.

